# Numerous mitochondrial DNA haplotypes reveal multiple independent polyploidy origins of hexaploids in *Carassius* species complex

**DOI:** 10.1002/ece3.3462

**Published:** 2017-11-04

**Authors:** Xiao‐Li Liu, Xi‐Yin Li, Fang‐Fang Jiang, Zhong‐Wei Wang, Zhi Li, Xiao‐Juan Zhang, Li Zhou, Jian‐Fang Gui

**Affiliations:** ^1^ State Key Laboratory of Freshwater Ecology and Biotechnology Institute of Hydrobiology Chinese Academy of Sciences University of the Chinese Academy of Sciences Wuhan China

**Keywords:** diploidization, ecological adaption, evolution, hexaploid, polyploidy, tetraploid

## Abstract

Evolutionary trajectory and occurrence history of polyploidy have been extensively studied in plants, but they remain quite elusive in vertebrates. Here, we sampled and gathered 4,159 specimens of polyploid *Carassius* species complex including 1,336 tetraploids and 2,823 hexaploids from a large geographic scale (49 localities) across East Asia, and identified a huge number of 427 diverse haplotypes of mitochondrial control region, in which 74 haplotypes with total occurrence frequency up to 75.498% were shared by hexaploids and tetraploids. Significantly, these diverse haplotypes were clustered into four major lineages, and many haplotypes of hexaploids and tetraploids were intermixed in every lineage. Moreover, the evolutionary trajectory and occurrence history of four different lineages were revealed by a simplified time‐calibrated phylogenetic tree, and their geographic distribution frequencies and haplotype diversity were also analyzed. Furthermore, lineage C and D were revealed to undergo population expansion throughout mainland China. Therefore, our current data indicate that hexaploids should undergo multiple independent polyploidy origins from sympatric tetraploids in the polyploid *Carassius* species complex across East Asia.

## 1 INTRODUCTION

Polyploidy, as a significant driving force on speciation and evolutionary success, has been extensively elucidated in many respects of polyploid plants including morphology, physiology, genomics, and ecology (Jiao et al., 2011; Leitch & Leitch, 2008; Soltis, Marchant, Van de Peer, & Soltis, 2015). Polyploids usually have wider ecological amplitude and stronger environmental adaptability than their ancestors (Brochmann & Elven, 1992), as polyploidy commonly introduces higher genomic complexity and more innovation (Doyle et al., 2008; Soltis, Liu, Marchant, Visger, & Soltis, 2014). Moreover, polyploidy consequences are frequently associated with reproduction mode transition (Gui & Zhou, 2010; Mei & Gui, 2015), and dramatic genome shifts after polyploidization have been recognized as an important mechanism in speciation and trait evolution (Van de Peer, Maere, & Meyer, 2009). In comparison with the commonness of polyploids in plants, polyploid species are rare in vertebrates (Choleva et al., 2012; Furlong & Pwh, 2004), but some taxonomic groups of reptiles, amphibians, and teleost fishes have passed through the bottleneck of polyploidy and formed stable polyploid species (Ficetola & Stöck, 2016; Gui & Zhou, 2010; Otto & Whitton, 2000; Schmid, Evans, & Bogart, 2015). Especially in some groups of fishes, different ploidy forms such as triploids, tetraploids, hexaploids, and even octoploids have been found (Arai & Fujimoto, 2013; Collares‐Pereira, Matos, Morgadosantos, & Coelho, 2013; Mei & Gui, 2015; Takada et al., 2010). However, evolutionary trajectory and ecological adaption of these polyploids remain elusive in vertebrates.

*Carassius* species complex with wide distribution across the Eurasian continent and neighboring islands (Abramenko, Nadtoka, Makhotkin, Kravchenko, & Poltavtseva, 2004; Hanfling, Bolton, Harley, & Carvalho, 2005; Jakovlic & Gui, 2011; Jiang et al., 2013; Li, Li, Zhang, Zhou, & Gui, 2014; Liousia, Liasko, Koutrakis, & Leonardos, 2008; Sakai, Iguchi, Yamazaki, Sideleva, & Goto, 2009; Toth, Varkonyi, Hidas, Meleg, & Varadi, 2005; Zhou & Gui, 2017) has been found to possess different ploidy forms including tetraploids, hexaploids, and even octaploids (Gao et al., 2012; Gui & Zhou, 2010; Jiang et al., 2013; Takada et al., 2010). Tetraploids with 100 chromosomes are diploidizated allotetraploids that reproduce by sexual reproduction (Luo, Stadler, He, & Meyer, 2007; Ohno, Muramoto, Christian, & Atkin, 1967), while hexaploids with about 150 chromosomes are able to reproduce by dual modes including unisexual gynogenesis and bisexual reproduction (Gui & Zhou, 2010; Gui & Zhu, 2012; Zhou, Wang, & Gui, 2000), and octaploids reproduce only via unisexual gynogenesis (Xiao et al., 2011; Zhu & Gui, 2007). Recently, an early allopolyploidy event leading to ancestral allotetraploids has been revealed, and a recurrent autopolyploidy from allotetraploids that results in allohexaploids has been elucidated in the *Carassius* species complex (Li, et al., 2014; Luo et al., 2014). Therefore, the wide geographic distribution, coexistence of different ploidy forms, and occurrence of repeated polyploidy events make the *Carassius* species complex an ideal system to investigate evolutionary trajectory and ecological adaption of polyploidy in vertebrates (Gao et al., 2017; Liu et al., 2017).

Mitochondrial DNA (mtDNA), as a cytoplasmic marker, had been extensively utilized to study origin and evolutionary history of unisexual or polyploid vertebrates including gynogenetic Amazon molly *Poecilia Formosa* (Dang, Xia, Xu, & Zhang, 2016), gynogenetic *Phoxinus eos‐neogaeus* (Angers & Schlosser, 2007), hybridogenetic *Poeciliopsis* (Quattro, Avise, & Vrijenhoek, 1992), hybridogenetic Australian carp gudgeon (Schmidt, Bond, Adams, & Hughes, 2011), kleptogenetic salamanders (Bi & Bogart, 2010; Robertson, Ramsden, Niedzwiecki, Fu, & Bogart, 2006), and parthenogenetic lizards (Hedges, Bezy, & Maxson, 1991). And, they were efficiently used to analyze genetic diversity and evolutionary implications of the polyploidy *Carassius* species complex in several different geographic populations (Apalikova, Eliseikina, Kovalev, & Brykov, 2008; Brykov et al., 2002; Gao et al., 2012; Jakovlic & Gui, 2011; Li & Gui, 2008; Takada et al., 2010; Wang et al., 2011). To further reveal evolutionary trajectory and ecological adaption of tetraploids and hexaploids in the *Carassius* species complex, a huge number of specimens were sampled throughout mainland China, and numerous mtDNA sequences including the newly obtained and previously reported sequences were used to perform a comprehensive investigation from a large geographic scale across East Asia.

## 2 METHODS

### 2.1 Sampling and specimen collection

A total of 3,105 individuals of the *Carassius* species complex were currently sampled from 34 locations through mainland China. And the other 1,054 sample data of *Carassius* species complex from 15 localities in East Asia were collected from previous reports (Luo et al., 2014; Takada et al., 2010). Details about all the samples, sampling sites, and references are given in Table 1. For currently sampled specimens, caudal fin was preserved in 100% ethanol for subsequent DNA extraction and sequencing, and the blood cells were fixed in 70% ethanol for ploidy determination as described (Jiang et al., 2013). For the previously reported samples, the ploidy forms were collected from previous reports (Luo et al., 2014; Takada et al., 2010), and the mitochondrial control region (CR) sequences were obtained from GenBank. All experiments in this research were performed according to the permit guidelines established by the Institute of Hydrobiology, Chinese Academy of Sciences, and the experimental protocols were approved by the animal care and use committee of Institute of Hydrobiology, Chinese Academy of Sciences.

**Table 1:** Specimen information of *Carassius* species complex used in this study

Code	Sampled locality	Abbreviation	Geographic coordinate	Sampled number	Tetraploid percentage (%)	Hexaploid percentage (%)	Geographic area
1	Hongze Lake, Sihong county	HZ	118.719°E, 33.291°N	126	93.65	6.35	Jing‐Hang Grand Canal of China
2	Gaoyou Lake, Gaoyou county	GY	119.348°E, 32.864°N	100	90	10	
3	Luoma Lake, Suyu district of Suqian	LM	118.182°E, 34.103°N	100	89	11	
4	Weishan Lake, Weishan county	WS	116.752°E, 35.113°N	80	76.25	23.75	
5	Dianchi, Chenggong district of Kunming	DC	102.736°E, 24.853°N	74	0	100	Upper Yangtze River of China
6	Puan, Puan county	PA	105.070°E, 25.538°N	96	0	100	
7	Fujiang, Hechuan district	FJ	106.227°E, 29.993°N	60	1.67	98.33	
8	Jialingjiang, Beibei district	JLJ	106.449°E, 29.826°N	60	1.67	98.33	
9	Yunan (Luo et al., 2014)	YN	102.852°E, 24.876°N	203	28.08	71.92	
10	Puan county, Guizhou (Luo et al., 2014)	GZ	104.960°E, 25.782°N	27	0	100	
11	Beimin Lake, Jinshi county	BM	111.886°E, 29.712°N	60	1.67	98.33	Middle Yangtze River of China
12	Shanbo Lake, Anxiang county	SB	112.041°E, 29.428°N	60	5	95	
13	Xihu Lake, Jinshi county	XH	111.934°E, 29.365°N	60	43.33	56.67	
14	Xiaoshui, Shuangpai county	XS	111.721°E, 25.899°N	172	1.16	98.84	
15	Dongting Lake, Xiangyin county	DT	112.693°E, 28.811°N	100	34	66	
16	Taibai Lake, Huangmei county	TB	115.828°E, 29.965°N	96	18.75	81.25	
17	Honghu Lake, Honghu county	HH	113.373°E, 29.821°N	80	86.25	13.75	
18	Dongting Lake, Hunan (Luo et al., 2014)	HN	112.584°E, 28.888°N	11	45.45	54.55	
19	Longgan Lake, Huangmei county	LG	116.041°E, 29.944°N	86	80.23	19.77	Lower Yangtze River of China
20	Poyang Lake, Duchang county	PY	116.301°E, 29.214°N	105	89.52	10.48	
21	Taihu Lake, Wuxi city	TH	120.183°E, 31.257°N	118	72.88	27.12	
22	Zhejiang (Luo et al., 2014)	ZJ	120.127°E, 30.126°N	44	52.27	47.73	
23	Chagan Lake, Qianguo county	CG	124.284°E, 45.270°N	100	9	91	Northeast of China
24	Jingbo Lake, Ningan county	JB	128.911°E, 43.854°N	100	6	94	
25	Xingkai Lake, Mishan city	XK	132.264°E, 45.228°N	100	0	100	
26	Suifen River, Suifenhe city	SF	131.115°E, 44.409°N	103	0	100	
27	Songhua Lake, Jiaohe city	SH	126.932°E, 43.603°N	100	2	98	
28	Songhuajiang, Haerbin city (Luo et al., 2014)	SHJ	128.457°E, 45.922°N	47	61.7	38.3	
29	Fangzheng county, Haerbin city (Luo et al., 2014)	FZ	128.829°E, 45.851°N	18	0	100	
30	Dawusong Lake, Heshuo county	DWS	87.222°E, 41.966°N	88	62.5	37.5	Northwest of China
31	Bositeng Lake, Heshuo county	BST	86.876°E, 41.942°N	100	47	53	
32	Tian'e Lake, Hejing county	TE	84.116°E, 42.919°N	100	13	87	
33	500 reservoir, Fukang city	R500	87.830°E, 44.180°N	90	0	100	
34	IrtySh River, Aletai district	IS	87.747°E, 47.393°N	77	0	100	
35	Wulungu Lake, Aletai district	WLG	87.123°E, 47.234°N	56	0	100	
36	Yili River, Gongliu county	YL	82.452°E, 43.597°N	46	0	100	
37	Lijiang, Xing'an county	LJ	110.344°E, 25.530°N	105	0	100	Upper Pearl River of China
38	Guangzhou city (Luo et al., 2014)	GD	113.264°E, 23.129°N	14	0	100	
39	Yellow River, Xingqing district of Yinchuan	YC	106.448°E, 38.387°N	105	0	100	Yellow River of China
40	Yellow River, Hubin district of Sanmenxia	SMX	111.154°E, 34.782°N	102	0	100	
41	Yellow River, Jiyuan city	JY	112.384°E, 34.923°N	100	0	100	
42	Honshu, Japan (Takada et al., 2010)	HO	139.669°E, 37.217°N	131	38.93	61.07	Main islands of Japan
43	Kyusyu, Japan (Takada et al., 2010)	KY	130.858°E, 32.491°N	22	27.27	72.73	
44	Shikoku, Japan (Takada et al., 2010)	SHK	133.385°E, 33.523°N	21	19.05	80.95	
45	Shibuta River, Tokyo (Takada et al., 2010)	SHB	139.703°E, 35.658°N	70	54.29	45.71	
46	LakeBiwa, Shiga Prefecture (Takada et al., 2010)	BI	136.167°E, 35.333°N	36	13.89	86.11	
47	Imba,River, Tokyo, Japan (Takada et al., 2010)	IM	140.123°E, 35.605°N	16	0	100	
48	Lake Kasumigaura, Tokyo (Takada et al., 2010)	KA	140.230°E, 36.080°N	5	0	100	
49	Ryukyus, Japan (Takada et al., 2010)	RY	128.946°E, 27.186°N	389	57.58	42.42	Ryukyus

### 2.2 Ploidy determination

High speed sorting flow cytometer FACSAriaTMIII (BD) was used to estimate ploidy levels of currently sampled specimens by measuring the relative DNA content of their fixed blood cells as described previously (Wei, Zhang, Zhang, Zhou, & Gui, 2003). Chicken blood cells with known DNA content of 2.5 pg/nucleus were used as an internally quantitative standard for each sample flow cytometry profile. The sampled blood cells of each sample were mixed with chicken blood cells and fixed in 70% precooled ethanol overnight at 4°C. The mixed cells were washed 2–3 times in 1× phosphate‐buffered saline and then resuspended in the solution including 0.5% pepsin and 0.1 M HCl. DNA was stained with propidium iodide solution (40 g/ml) for 1–3 hr at room temperature in the dark. Each sample contained three repeats, and each repeat was measured at least 10,000 cells. DNA contents for each sample were measured by a formula in which the PI fluorescence intensity ratio of the mean of blood cells from each individual to the mean of blood cells from chicken was multiplied by the known mean DNA content (2.5 pg) of chicken blood cells as described (Wei et al., 2003) and was generated automatically by the flow cytometer. As reported previously (Jiang et al., 2013), the individuals with average DNA contents of about 3.64 pg/N ± 0.12 were generally detected as tetraploids, whereas the individuals with near 5.42 pg/N ± 0.198 were identified as hexaploids.

### 2.3 Mitochondrial DNA sequencing and common sequence selection

Whole DNA was extracted from fin clips by DNeasy Blood & Tissue Kit (QIAGEN) following the manufacturer's protocol. We used the following primers L15923c (5′‐TTAAAGCATCGGTCTTGTAA‐3′) and H16500d (5′‐GCCCTGAAATAGGAACCAGA‐3′) (Jiang et al., 2013) to amplify mitochondrial CR sequences. Purified PCR products were directly used for bidirectional Sanger sequencing on PRISM 3700 (ABI) via primers L15923c and H16500d.

The corresponding common sequences with the newly generated mitochondrial CR s were selected from previously reported data (Luo et al., 2014; Takada et al., 2010) and were aligned with all of them via multiple alignments using MEGA7.0 (Kumar, Stecher, & Tamura, 2016). At last, the common sequences ranging from 298 to 353 bp were used for subsequent analyses.

### 2.4 Sequence and data analyses

Sequence alignments and information of haplotypes were identified using MEGA7.0 (Kumar et al., 2016). Haplotypes were generated by DnaSP 5.10 (Librado & Rozas, 2009) software and then arranged according to their frequency for easy reading. Haplotype diversity was evaluated using Arlequin version 3.5 (Excoffier & Lischer, 2010). Phylogenetic analysis of haplotypes was conducted using Bayesian inference (BI) in MRBAYES 3.1.2 (Ronquist & Huelsenbeck, 2003) and maximum‐likelihood (ML) in RA_X_ML (Stamatakis, Hoover, & Rougemont, 2008). HKY + I + G was selected as the best‐fit model of evolution by MODELTEST version 3.7 (Posada & Crandall, 1998). For BI tree, four independent Markov chain Monte Carlo (MCMC) chains were simultaneously run for 20,000,000 generations with sample frequency of 1,000 generations. The first 25% of the trees were discarded as burn‐in, and the remaining tree samples were used to generate a consensus tree. For ML tree, nodal support value was assessed from 100 nonparametric bootstrap replicates. In addition, to investigate the relationship between haplotypes, a network was built by TCS 1.21 (Clement, Posada, & Crandall, 2000).

To estimate divergence times in *Carassius* species complex, an uncorrelated relaxed molecular clock approach was implemented in Beast 1.7.5 (Drummond & Rambaut, 2007). HKY + I + G was selected as the best‐fit model of evolution by MODELTEST software of version 3.7 (Posada & Crandall, 1998). The divergence time (11.11–9.14 million years ago [Mya]) (Gao et al., 2012) between *Cyprinus carpio* and *Carassius* species complex was used as the calibration. The Markov chain Monte Carlo (MCMC) analyses were run for 100,000,000 generations with sample frequency of 1,000 generations. Tracer v1.5 (http://tracer-1-5.software.informer.com/1.5/) was used to ensure adequate mixing of the MCMC with effective sample sizes (ESS) above 100. The plausible trees were summarized in the maximum clade credibility (MCC) tree after discarding first 40% of sampled generations by Tree Annotator v1.7.5, and then the results were visualized and edited in FigTree 1.4 (http://beast.bio.ed.ac.uk/FigTree).

Historical demographic/spatial expansions of *Carassius* species complex were investigated by two approaches. First, Tajima's *D* (Tajima, 1989) and Fu's *Fs* (Fu, 1997) statistics were calculated. Second, pairwise mismatch distributions (Schneider & Excoffier, 1999) were used to detect the demographic history of *Carassius* species complex. When the neutrality test values were negative and significant (*p* < .05), and the mismatch distribution curve was unimodal, the lineage was thought to fit the sudden expansion model. Both the two approaches were performed by Arlequin 3.5 (Excoffier & Lischer, 2010). Moreover, the expansion time was estimated by the equation τ = 2*ut* (Nei & Tajima, 1981; Rogers & Harpending, 1992), in which *u* is the mutation rate per sequence and per generation. The value of *u* was calculated from *u* = 2μ*k*, where μ is the mutation rate per nucleotide, and *k* is the number of nucleotides in the analyzed fragment. The approximate time of expansion was calculated by multiplying *t* by the generation time (1 year; Luo et al., 2014). The average substitution rate of 2% site^−1^ Myr^−1^ (Meyer, 1993) was used for mitochondrial CR haplotypes of the *Carassius* species complex.

## 3 RESULTS

### 3.1 Ploidy distribution of *Carassius* species complex across East Asia

**Figure 1:**
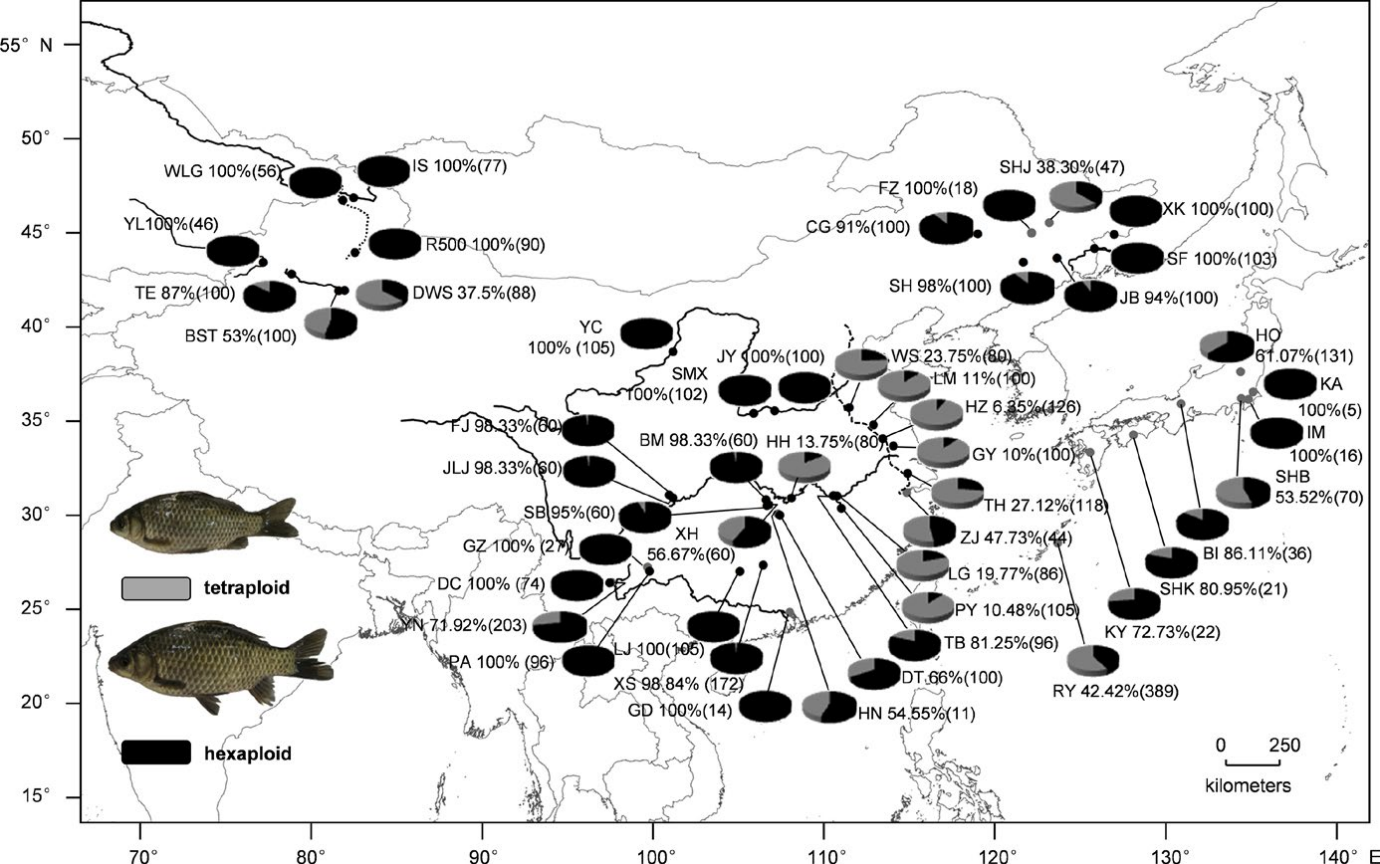
Specimen and ploidy distribution of *Carassius* species complex across East Asia. Detail information of the sampled and gathered populations is in Table 1. The proportions of hexaploids and tetraploids are indicated by the sizes of black and gray pie charts, respectively. The hexaploid percentages and the specimen numbers are given nearby and in the brackets

A total of 4,159 specimens of *Carassius* species complex were gathered from 49 localities across East Asia. As shown in Table 1 and Figure 1, 3,105 specimens comprising 894 tetraploids and 2,211 hexaploids are currently sampled from 34 localities throughout mainland China reported recently (Liu et al., 2017), 364 specimens including 114 tetraploids and 250 hexaploids are from seven localities of mainland China reported previously (Luo et al., 2014), and 690 individuals containing 328 tetraploids and 362 hexaploids come from eight localities of Japan main islands and Ryukyus as described (Takada et al., 2010). In the 49 populations, there are 17 all‐hexaploid populations and 32 sympatric populations of both hexaploids and tetraploids, whereas there is no one all‐tetraploid population (Figure 1). And even in the 32 sympatric populations of both hexaploids and tetraploids, most of them (19 of 32) consist of high frequency of hexaploids, while only a few populations (13 of 32) are tetraploid‐biased. In terms of different drainage systems or geographic areas, all‐hexaploids and highly biased‐hexaploid populations are predominant in northeast of China (except SHJ), northwest of China (except DWS), Yellow River basin, upper Yangtze River, upper Pearl River, and two localities of Japan main islands, whereas tetraploid‐biased populations are mainly distributed in Middle/Lower Yangtze River basin, Jing‐Hang Grand Canal and Ryukyus. As reported recently (Liu et al., 2017), hexaploids have wider geographic distribution than tetraploids in *Carassius* species complex throughout East Asia.

### 3.2 Identification of diverse mtDNA CR haplotypes

Subsequently, a total of 316 various mtDNA CR haplotypes were identified from the newly sequenced 3,105 specimens, and 57 and 72 haplotypes were, respectively, retrieved from two previous reports by Luo et al. (2014) and Takada et al. (2010). As 18 haplotypes in the newly identified 316 haplotypes were same to that reported by Luo et al. (2014), a total of 427 various mtDNA CR haplotypes (Table S1) were gathered at last. The length variations of 427 mtDNA CR haplotypes ranged from 298 to 353 bp, and contained 154 variable positions, of which 109 were potentially parsimony informative (Fig. S1). Among the 427 haplotypes, there were 74 haplotypes to share by hexaploids and tetraploids, in which each haplotype frequency ranged from 14.378% to 0.048%, and their total occurrence frequency was high up to 75.498%. There were 217 haplotypes and 136 haplotypes to be detected only in hexaploids or in tetraploids, but their total occurrence frequency was 18.755% and 5.747%, respectively, in which each haplotype frequency ranged from 1.467% to 0.024% in hexaploids and from 0.457% to 0.024% in tetraploids. Moreover, 130 haplotypes and 98 haplotypes were respectively detected from only one hexaploid or one tetraploid individual (Table S2).

### 3.3 Matrilineal genealogy of mtDNA CR haplotypes

**Figure 2:**
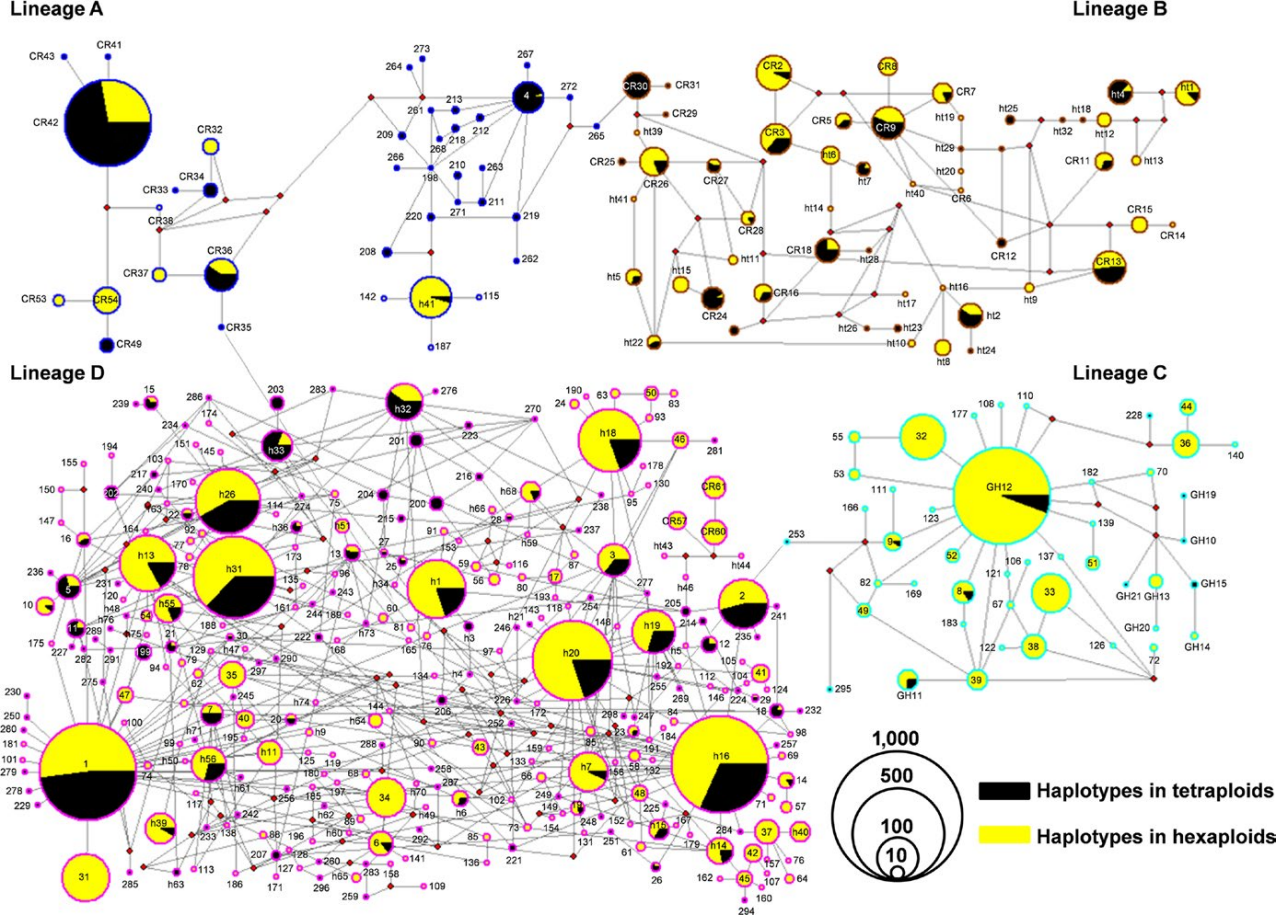
Network of 427 mitochondrial DNA control region haplotypes identified from the polyploid *Carassius* species complex. Circles represent different haplotypes and their corresponding occurrence frequency in all sampled populations. Border colors stand for different lineages A, B, C, and D. Black and yellow inside the circle indicate the percentage of tetraploid and hexaploid, respectively. Haplotype codes are denoted inside or beside the circles. Solid red dots represent unsampled or predicted haplotypes

Based on the 427 diverse mtDNA CR haplotypes, a network was constructed by TCS 1.21 (Clement et al., 2000) to investigate their matrilineal genealogy. As shown in Figure 2, four major lineages are clustered, and haplotypes of hexaploids and tetraploids are intermixed in all of the four lineages. Lineage A includes 39 haplotypes, in which four haplotypes are shared by hexaploids and tetraploids, and eight and 27 haplotypes exist only in hexaploids or tetraploids respectively. The highest frequency haplotype in lineage A is CR42 (4.4%; Figure 2 and Table S2) that distributes only in Ryukyus (Table S1), and h41 is the most widely distributed haplotype in lineage A, which occurs in seven local populations of China including GY, YN, XK, LJ, GD, SMX, JY (Table S1). Lineage B includes 54 haplotypes that distribute in Japan main islands and Ryukyus, in which 19 haplotypes are shared by hexaploids and tetraploids, and 21 and 14 haplotypes exist only in hexaploids or tetraploids, respectively. CR2 is the highest frequency haplotype with only 0.745% in lineage B (Figure 2 and Table S2). A total of 45 haplotypes are grouped into lineage C, where four are shared by hexaploids and tetraploids, and 34 and seven haplotypes exist only in hexaploids or tetraploids, respectively. In lineage C, the highest frequency haplotype is GH12 (9.930%; Figure 2) that distributes in most newly sampled populations of China (22/34; Table S1), and other haplotypes in this lineage are derived from GH12 due to the star‐like phylogenetic relationship (Figure 2). Lineage D is the largest and includes 289 haplotypes, in which 47 haplotypes are shared by hexaploids and tetraploids, and 154 and 88 haplotypes exist only in hexaploids and tetraploids, respectively. Haplotype CRH1 with the highest frequency occurrence (14.378%; Figure 2) extensively coexists in both hexaploids and tetraploids of all newly sampled populations throughout mainland China except PA and JLJ (Table S1), implicating that CRH1 might be one of the ancestor haplotypes of *Carassius* species complex. Significantly, the first 10 major haplotypes among 427 haplotypes are shared by both hexaploids and tetraploids (Table S2), and they also represent a star‐like phylogenetic relationship around them except CR42 in lineage A.

### 3.4 Phylogenetic relationship and evolutionary history of mtDNA CR haplotypes

**Figure 3:**
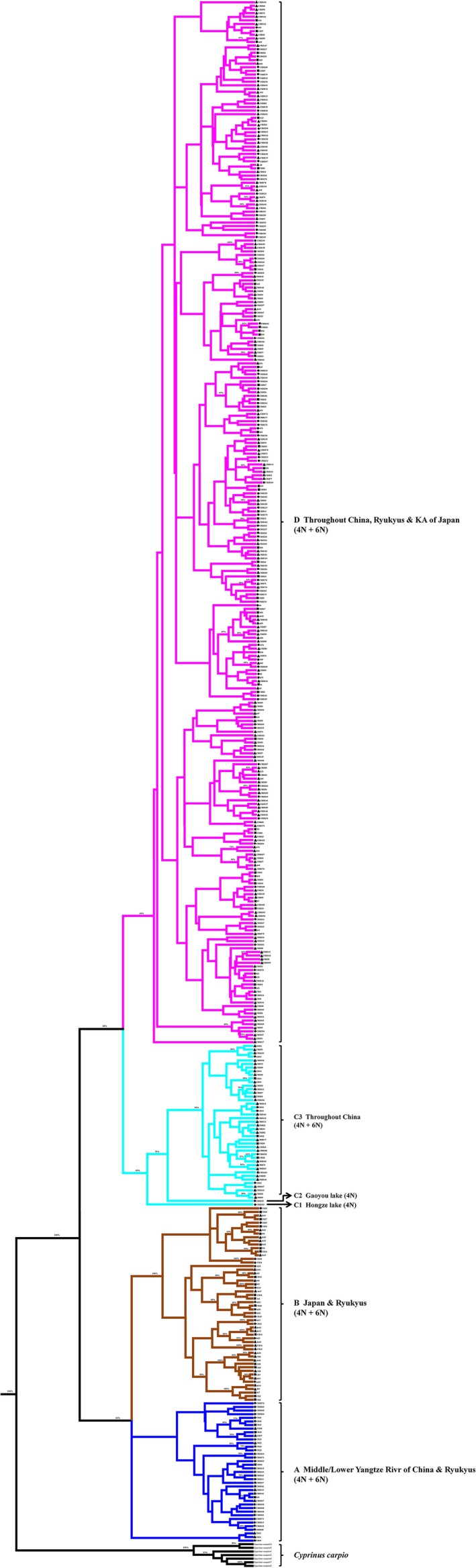
Bayesian tree of 427 mitochondrial DNA (mtDNA) control region (CR) haplotypes identified from the polyploid *Carassius* species complex. Bayesian posterior probabilities (BPP) of >50% are shown around nodes. Major lineages are shown by different colors. Lineage A, B, C, and D are exhibited in blue, brown, green, and pink, respectively. Solid squares indicate shared haplotypes between tetraploids and hexaploids. Solid triangles and circles represent haplotypes only detected from hexaploids and tetraploids, respectively. 4N and 6N are the abbreviation of tetraploids and hexaploids, respectively. Seven mtDNA CR haplotypes from *Cyprinus carpio* are used as outgroup

To pursue the phylogenetic relationship of polyploidy occurrence and divergence evolution in *Carassius* species complex, phylogenetic BI tree and ML tree were also constructed according to these diverse CR haplotypes. And only BI tree was given in Figure 3, as both ML and BI tree were well supported each other and showed the same topology. Similar to haplotype network, the diverse 427 mtDNA CR haplotypes were also clustered into four major lineages with obvious geographical distribution differences, and haplotypes of hexaploids and tetraploids were intermixed in each lineage as well.

**Figure 4:**
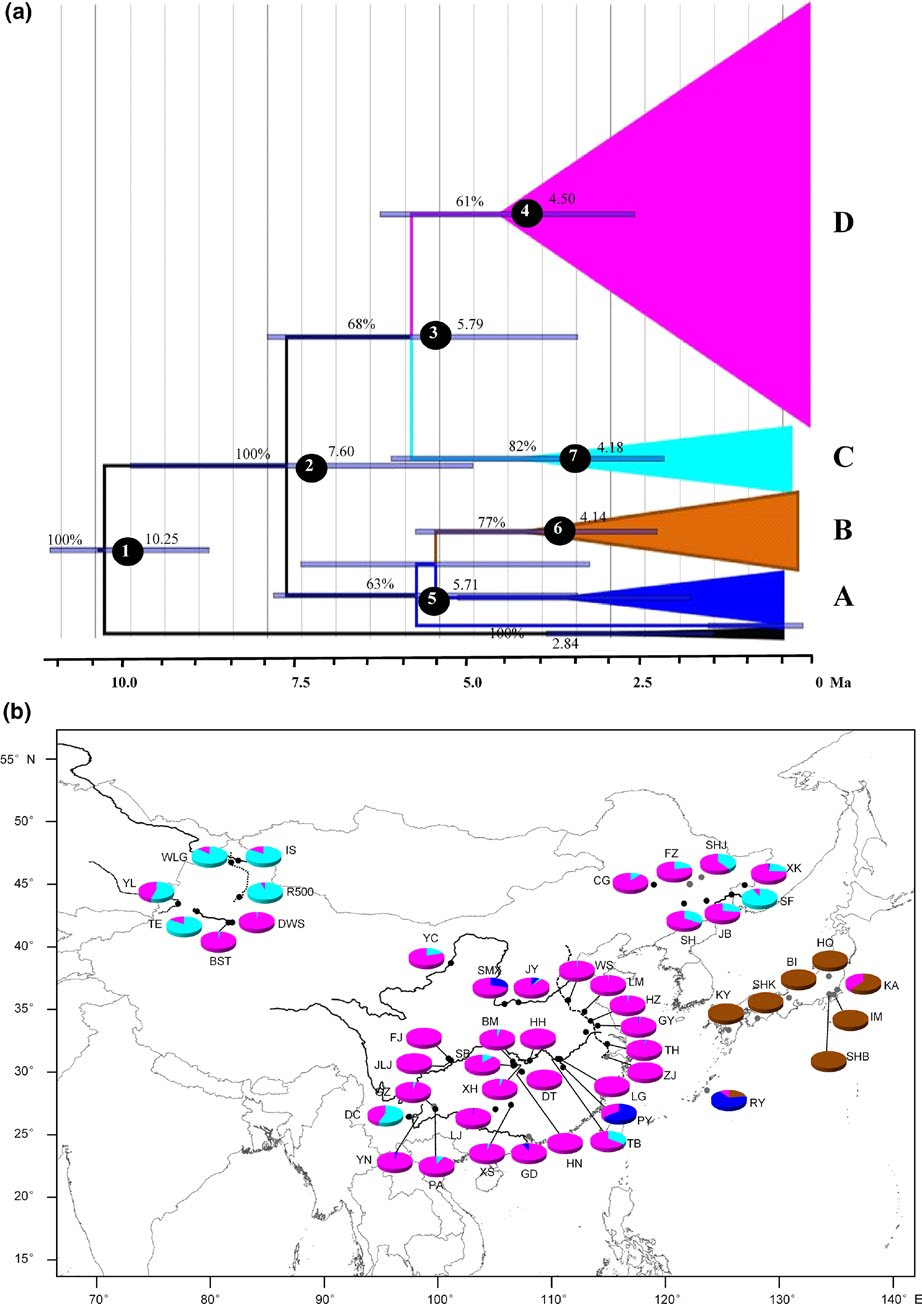
Simplified time‐calibrated phylogeny tree of four major haplotype lineages (a) and their geographical distribution (b). Color in each lineage is corresponding to the Bayesian tree in Figure 3. Values above branches indicate Bayesian posterior probabilities, and divergence times are shown near each node. The numbers in black circles indicate estimated divergence time listed in Table S3. Haplotypes of *Cyprinus carpio* are used as outgroup

Moreover, a simplified time‐calibrated phylogenetic tree of the diverse mtDNA CR haplotypes was constructed (Figure 4a), and the relative frequencies of four different lineages in all 49 local populations were presented (Figure 4b). As shown in Figure 4, within *Carassius* species complex, A and B lineages split from C and D lineages early at about 7.60 Mya, then lineage A and lineage B diverge around 5.71 Mya, and lineage C and lineage D divide at about 5.79 Mya (Figure 4a, Table S3). The haplotypes clustered in lineage A were mainly detected in some locations of central/southern China including Yangtze River basin, Yellow River basin, Pearl River basin, Jing‐Hang Grand Canal, and Ryukyus island, and several haplotypes were also observed at one point (XK) in northeast of China. Haplotypes of lineage B were completely distributed in Japan and Ryukyus islands. Haplotypes of lineage C were distributed throughout mainland China, in which the occurrence frequencies were relatively higher in northern China than in central/southern China. Haplotypes of lineage D were scattered in all populations throughout mainland China, and most of them occupied relatively dominant frequencies of more than 90% in central/south populations (22/27), in which there were seven local populations with 100% haplotypes of lineage D. Significantly, the dominant distribution was also detected in BST and DWS of northwest China, and a few of haplotypes (five) were also found in KA of Japan and Ryukyus.

### 3.5 Population expansion possibility of different mtDNA haplotype lineages

**Figure 5:**
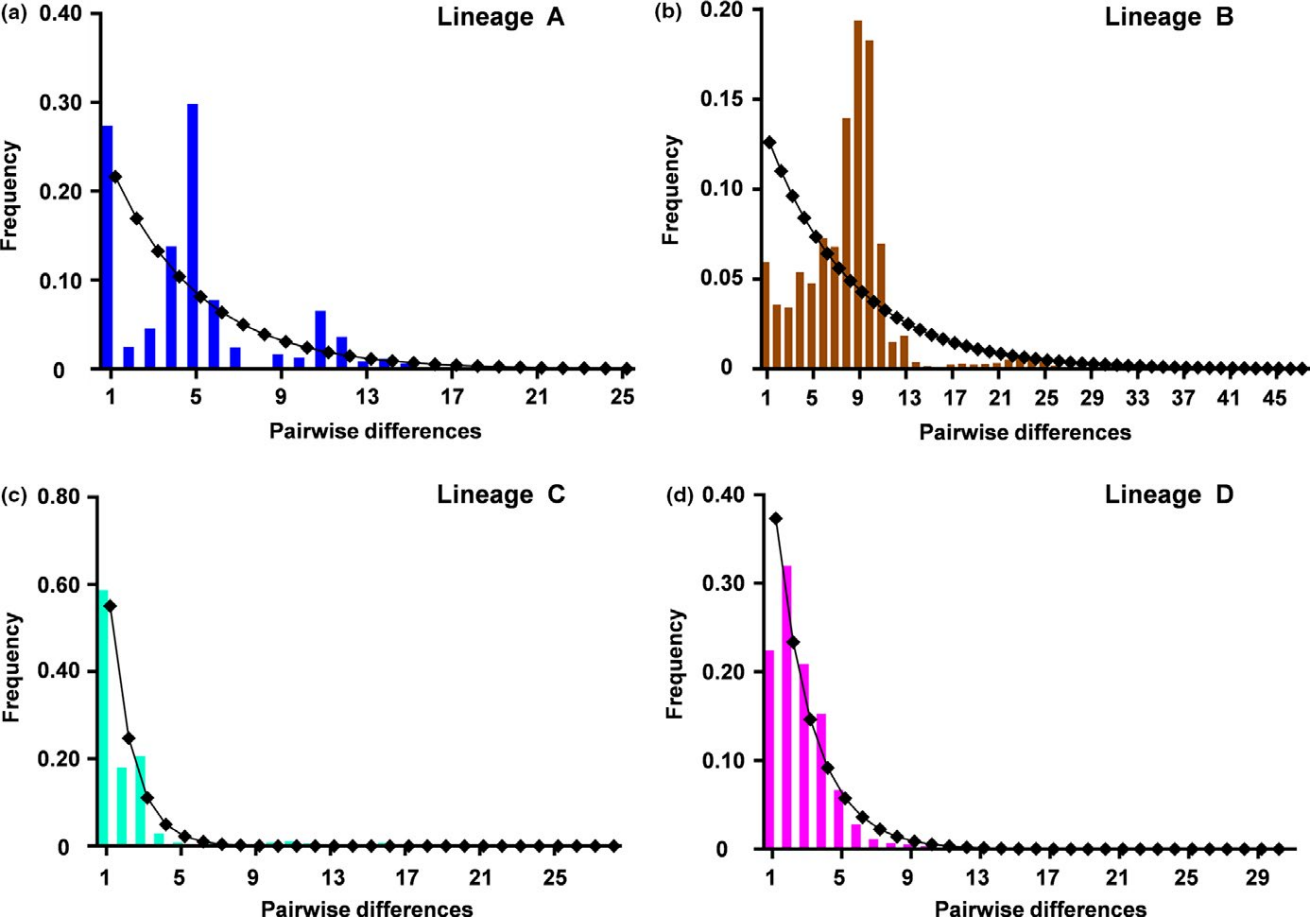
Mismatch distributions for each lineage of *Carassius* species complex. The *X*‐axis indicates the number of pairwise differences between compared haplotypes. The *Y*‐axis is the frequency for each value. Histograms indicate the observed frequencies of pairwise divergences among haplotypes, and the lines denote the expectation under the model of population expansion. Color of each histogram is corresponding to color of lineage in Figure 3. (a–d) Mismatch distributions for the lineage A, B, C, and D, respectively

Based on mtDNA CR haplotype sequences for each lineage, we calculated the statistical data about neutrality test and mismatch analysis by the reported approaches (Excoffier & Lischer, 2010; Fu, 1997; Schneider & Excoffier, 1999; Tajima, 1989), and thereby evaluated the historical demography and population expansion possibility in *Carassius* species complex across East Asia. As shown in Table 2 and Figure 5, for lineage A, Tajima's *D* and Fu's *Fs* tests are positive and not significant (Table 2), and mismatch distribution analysis shows bimodal distribution of pairwise differences (Figure 5a), indicating that lineage A rejects the hypothesis of sudden expansion (Table 2). For lineage B, although mismatch analysis reveals approximately unimodal distribution of pairwise differences (Figure 5b), Tajima's *D* and Fu's *Fs* tests are positive and not significant (Table 2), implying that lineage B also rejects the sudden expansion model. For lineage C and lineage D, Tajima's *D* and Fu's *Fs* tests are negative and significant (Table 2), and mismatch distribution analysis exhibits a unimodal distribution of pairwise differences (Figure 5c,d), showing that lineage C and lineage D do not reject the sudden expansion model. Therefore, these data suggest that lineage C and lineage D distributed throughout mainland China might have undergone population expansion, and the expansion time might occur at about 890–940 and 91,000–108,000 years ago, respectively (Table 2), whereas other two lineages might not encounter population expansion.

**Table 2:** Statistical data of neutrality tests and mismatch analyses based on mtDNA control region haplotype sequences for each lineage

Lineage	Neutrality test		Mismatch analysis			
	Tajima's *D* (*p*‐value)	Fu's *Fs* (*p*‐value)	SSD (*p*‐value)	Raggedness index (*p*‐value)	Tau	Expansion time (Mya)
Lineage A	0.1304 (.6350)	8.5978 (.9040)	0.1140 (.1200)	0.1522 (.0100)	40.6172	–
Lineage B	−0.2701 (.442)	−1.2232 (.4870)	0.0083 (.2000)	0.0077 (.0800)	8.1270	–
Lineage C	−2.2204 (.0000)	−24.4773 (.0000)	0.4288 (.0000)	0.0657 (1.0000)	0.025	0.00089–0.00094
Lineage D	−1.9321 (.0010)	−24.2047 (.0060)	0.0015 (.7500)	0.0103 (.8700)	2.5684	0.091–0.108

## 4 DISCUSSION

Our current studies had identified and gathered numerously diverse mtDNA CR haplotypes (427 haplotypes) of polyploid *Carassius* species complex from a huge number of specimens (4,159 specimens) and a large geographic scale (49 localities) across East Asia (Figure 1 and Table 1), and analyzed each haplotype variation and occurrence frequency in hexaploids or in tetraploids, respectively (Fig. S1, Table S2). Subsequently, these diverse haplotypes were clustered into four major lineages, and many haplotypes of hexaploids and tetraploids were intermixed in every lineage (Figures 2 and 3). Significantly, the first 10 haplotypes with high occurrence frequency were found to share by both hexaploids and tetraploids, and their occurrence frequency was high up to 53.017% (Table S2). Moreover, a simplified time‐calibrated phylogenetic tree revealed evolutionary history of four different lineages (Figure 4a), and their geographic distribution frequencies (Figure 4b). And the lineage C and D that distribute throughout mainland China were revealed to undergo population expansion by neutrality test, mismatch analysis (Figure 5 and Table 2). Therefore, these data suggest that multiple independent polyploidy origins should have occurred in hexaploids of the polyploid *Carassius* species complex across East Asia, as numerous CR haplotypes of hexaploids and tetraploids are intermixed in each lineage, and many CR haplotypes especially high occurrence frequency haplotypes are shared by both hexaploids and tetraploids (Figures 2 and 3).

Previous studies have proposed that an early allopolyploidy event might lead to tetraploids, and an autopolyploidy event might result in hexaploids in the polyploid *Carassius* species complex (Li, Zhang, Li, et al., 2014; Luo et al., 2014). The current studies further help us understand their evolutionary history and trajectory. As shown in Figures 2 and 3, in the four lineages revealed by diverse 427 mtDNA CR haplotypes, most of the haplotypes in hexaploids and tetraploids are intermixed in the same lineages, and numerous haplotypes are shared by both hexaploids and tetraploids. Thus, hexaploids might at least have four independent origins across Central and East Asia, and might originate from sympatric tetraploids via autopolyploidy. Lineage A and lineage B were split with lineage C and lineage D at about 7.60 Mya, in which lineage A was further separated with lineage B at about 5.71 Mya, and lineage C was divided with lineage D at approximate 5.79 Mya. These data suggest that lineage A should be the oldest lineage in the polyploid *Carassius* species complex, and Yangtze River basin might be the potential origin center, because lineage A mainly distributed in Yangtze River basin (Figure 4), and mtDNA CR haplotype diversity was higher in Yangtze River basin than in other areas examined (Fig. S2). Moreover, lineage C and lineage D might derive from Yangtze River basin, in which lineage C might thereby diffuse to northwest China and northeast China, and lineage D might extend throughout mainland China via population expansion (Figure 5 and Table 2). Actually, these deduced evolutionary history and trajectory are basically consistent with the time record of *Carassius* species fossil in Pliocene epoch (5.3–2.6 Mya) discovered in north of China (Yushe, Shanxi province) (Liu & Su, 1962).

Significantly, several unusual area existences of mtDNA CR haplotypes further revealed certain extent effects of human activities on geographic distribution of the polyploid *Carassius* species complex, as they have been important commercial and aquaculture fishes (Zhou, Wang, Wang, & Gui, 2017). Obviously, the exceptional dominant distribution of lineage D haplotypes in BST and DWS of northwest China might be resulted from artificial introduction, as other locations of northwest China were revealed to mainly contain haplotypes of lineage C, and multiple introductions in BST and DWS were recorded from middle and lower basins of the Yangtze River (Peng, Xue, Guo, & Xue, 2008). In Ryukyus island, the examined population was mainly consisted of lineage A haplotypes, and a few of lineage B and lineage D haplotypes were also detected, indicating that the Ryukyus population of *Carassius* species complex might be artificially introduced from China and Japan, respectively, as suggested previously (Takada & Tachihara, 2009; Takada et al., 2010). In addition, a few haplotypes of lineage A observed in XK of northeast China and only two haplotypes of lineage D detected in KA of Japan might be resulted from human activities (Gao et al., 2012).

Diploidization process after polyploidy has been suggested to be the driving force of recurrent polyploidy (Soltis et al., 2015; Wendel, 2015). After autopolyploidy from sympatric tetraploids, the extant hexaploids in *Carassius* species complex may be entering an evolutionary trajectory of diploidization or have been on the diploidization process, as many phenomena similar to normal sexual diploid species have been revealed in some strains of hexaploid *Carassius gibelio*, such as normal meiosis completion, multiple modes of unisexual gynogenesis and sexual reproduction, and extra microchromosomes for male determination (Li et al., 2016; Zhang et al., 2015). However, recurrent polyploidy is not ceased, as octaploids have been also detected in some natural habitats occasionally (Table 1) (Liasko et al., 2010; Xiao et al., 2011). Therefore, the wide geographic distribution, coexistence of different ploidy forms and occurrence of repeated polyploidy events make the polyploid *Carassius* species complex an intriguing system to investigate evolutionary genetics and ecological genetics of vertebrates.

## CONFLICT OF INTEREST

None declared.

## AUTHORS CONTRIBUTION

JFG, LZ, ZWW, FFJ, and XLL collected samples, XJZ, ZL, and XLL performed the experiments, XLL and XYL analyzed the data, JFG and XLL prepared the data and wrote the manuscript. All authors read and approved the final manuscript.
