# Supplementary figures and images for: Numerous mitochondrial DNA haplotypes reveal multiple independent polyploidy origins of hexaploids in Carassius species complex
Source: Ecol Evol. 2017 Nov 4;7(24):10604–15. doi: 10.1002/ece3.3462 (PMC5743492; doi:10.1002/ece3.3462)

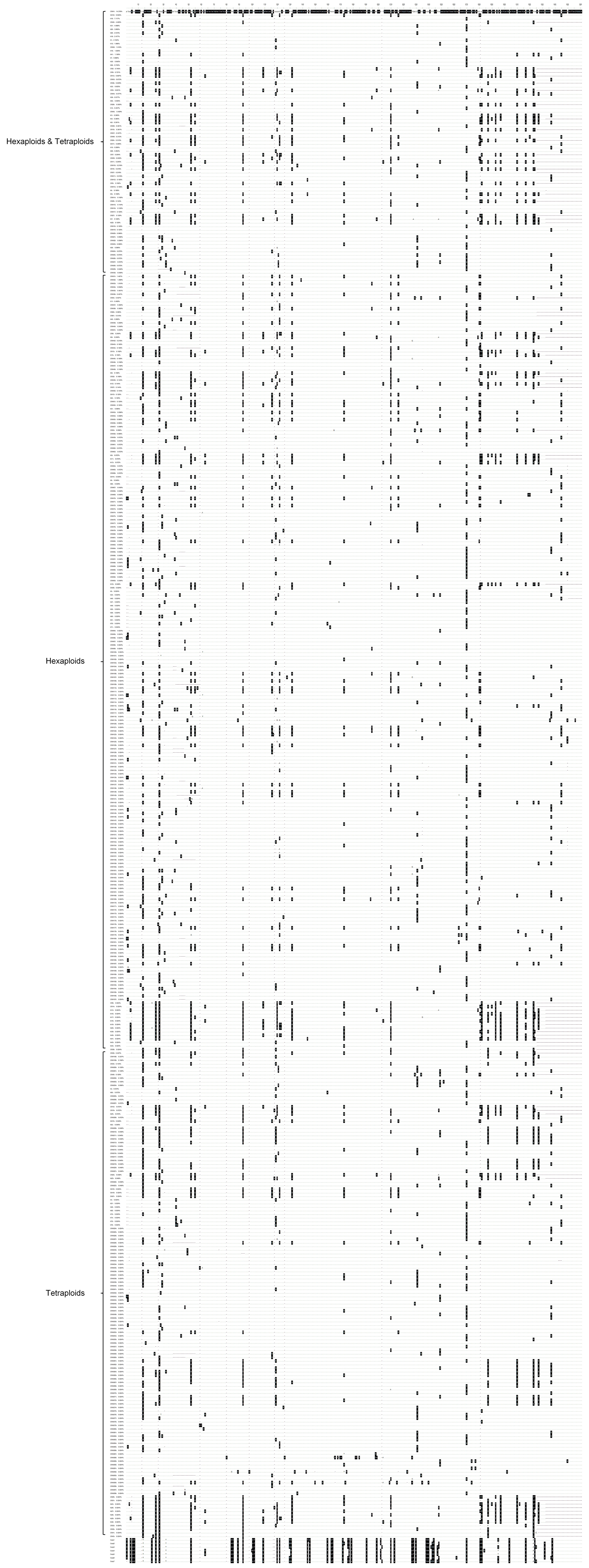

Supplement: Supplementary file 1 [file ECE3-7-10604-s001.tif]

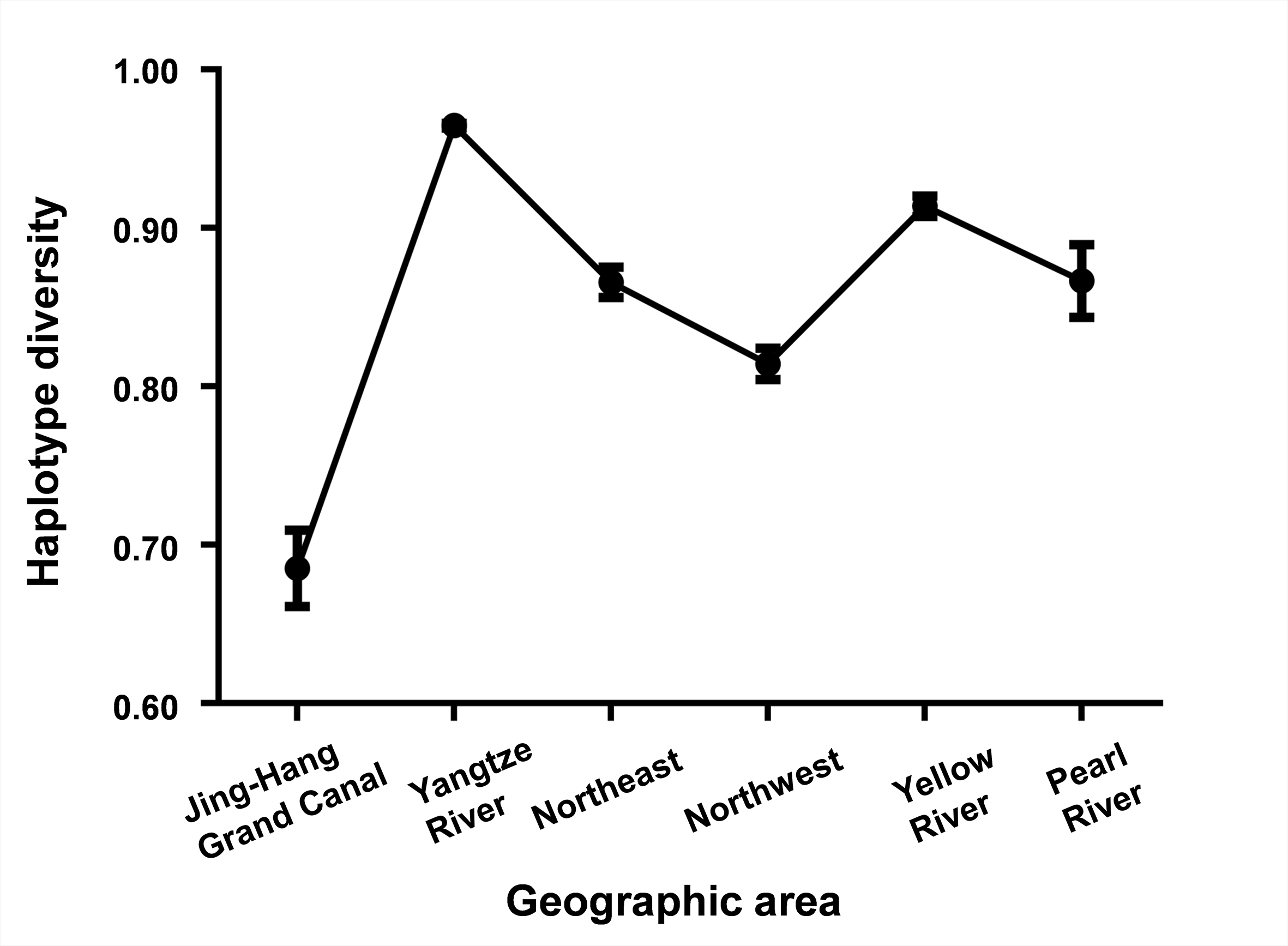

Supplement: Supplementary file 2 [file ECE3-7-10604-s002.tif]
