# Supplementary material for: Numerous mitochondrial DNA haplotypes reveal multiple independent polyploidy origins of hexaploids in Carassius species complex
Source: Ecol Evol. 2017 Nov 4;7(24):10604–15. doi: 10.1002/ece3.3462 (PMC5743492; doi:10.1002/ece3.3462)
Supplement: Supplementary file 4 [file ECE3-7-10604-s004.doc]

Electronic Supplementary Material (ESM) for:

**Numerous mtDNA haplotypes reveal multiple independent polyploidy origins of hexaploids in *Carassius* species complex**

**Xiao-Li Liu, Xi-Yin Li, Fang-Fang Jiang, Zhong-Wei Wang, Zhi Li, Xiao-Juan Zhang, Li Zhou, Jian-Fang Gui^1^**

**^1^**State Key Laboratory of Freshwater Ecology and Biotechnology, Institute of Hydrobiology, Chinese Academy of Sciences, University of the Chinese Academy of Sciences, Wuhan 430072, Hubei, China

**^1^Corresponding author:** Jian-Fang Gui, Institute of Hydrobiology, Chinese Academy of Sciences, Wuhan 430072, China, Tel: +86-27-68780707, Fax: +86-27-68780123, E-mail: [jfgui@ihb.ac.cn](mailto:jfgui@ihb.ac.cn)

**Supplementary figures**

**
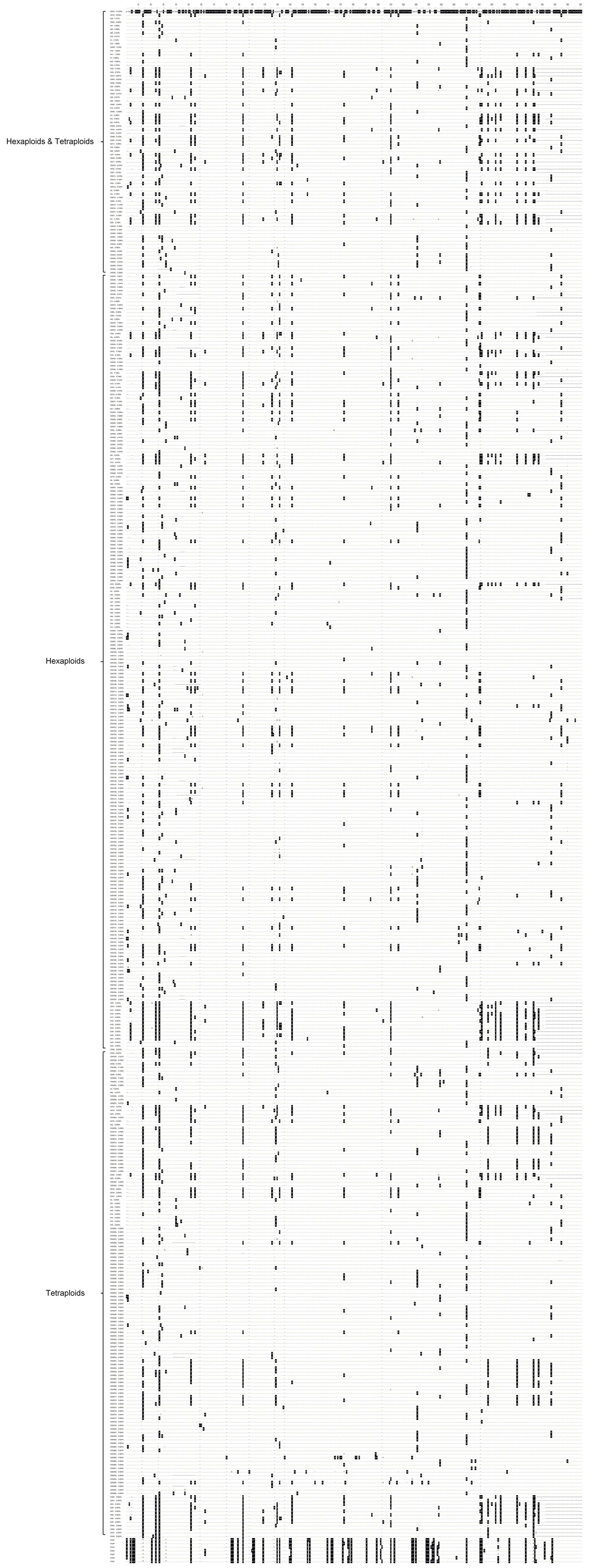
**

**Fig. S1.** Nucleotide sequences of the identified 427 mtDNA CR haplotypes. Gaps (-) are introduced to optimize identity. The occurrence frequencies are shown behind the name of each haplotype.


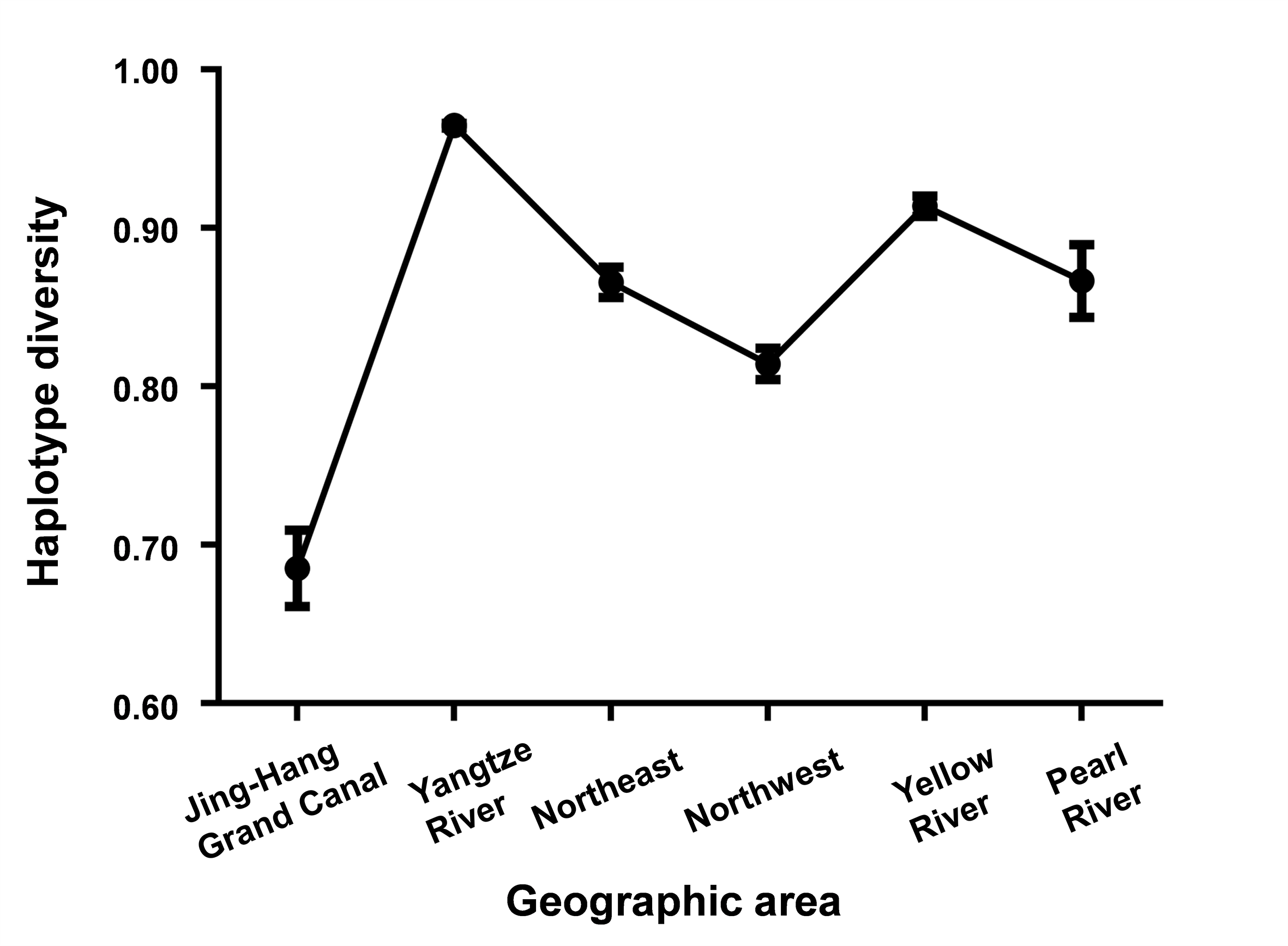


**Fig. S2.** Haplotype diversity of *Carassius* species complex mtDNA CR haplotypes in 6 different geographic areas. The X-axis shows geographic areas of the sampled locations, and Y-axis indicates value of haplotype diversity.

**Supplementary tables**

**Table S3**

Estimated mean divergence time (Mya) and 95% highest posterior density (HPD) range of *Carassius*

species complex. Node numbers are corresponding to those given besides the branches in Figure 4.

| Node number | Mean divergence time (Mya) | 95% HPD range (Mya) |
| --- | --- | --- |
| 1 | 10.25 | 8.73-11.05 |
| 2 | 7.60 | 4.89-9.88 |
| 3 | 5.79 | 3.36-7.87 |
| 4 | 4.50 | 2.53-6.24 |
| 5 | 5.71 | 3.36-7.79 |
| 6 | 4.14 | 2.21-5.71 |
| 7 | 4.18 | 2.10-6.07 |
